# A comparative study on the efficacy of ultrasound-guided interscalene brachial plexus block combined with modified superficial cervical plexus block in proximal humerus fracture surgery

**DOI:** 10.1097/MD.0000000000046267

**Published:** 2026-05-12

**Authors:** Gang Liu, Xiaoxuan Du, Lei Gao, Wei Wang, Feng Song

**Affiliations:** a Department of Anesthesiology, The Sixth Affiliated Hospital of Xinjiang Medical University, Urumqi, Xinjiang, China.

**Keywords:** anesthetic efficacy, interscalene brachial plexus, modified superficial cervical plexus, nerve block, proximal humerus

## Abstract

**Background:**

To compare the anesthetic efficacy and safety of ultrasound-guided interscalene brachial plexus block (ISBPB) combined with a modified superficial cervical plexus block (SCPB) versus the conventional combination in patients undergoing surgical fixation of proximal humerus fractures.

**Methods:**

In this prospective, randomized controlled study, 60 patients undergoing unilateral proximal humerus fracture surgery between January and October 2024 were enrolled. Participants were randomly assigned to either the modified group (receiving ultrasound-guided ISBPB combined with a modified SCPB) or the control group (receiving ultrasound-guided conventional ISBPB combined with a standard SCPB). The onset time of anesthesia, block quality, incidence of diaphragmatic paralysis, Horner syndrome, nerve injury, and patient satisfaction were recorded and compared between groups.

**Results:**

There was no statistically significant difference in the overall quality of nerve block between the 2 groups (*P* > .05). However, compared to the control group, the modified group showed a slightly prolonged onset time of anesthesia (*P* < .05), but a significantly lower incidence of diaphragmatic paralysis, Horner syndrome, and nerve injury (all *P* < .05). Furthermore, patient satisfaction scores were significantly higher in the modified group (*P* < .05).

**Conclusion:**

Ultrasound-guided ISBPB combined with a modified SCPB provides effective anesthesia for proximal humerus fracture surgery, while significantly reducing the incidence of block-related complications. This approach may be more favorable for postoperative recovery.

## 1. Introduction

Proximal humeral fractures are a common type of upper extremity trauma, characterized clinically by substantial tissue injury, significant bleeding, and severe pain. Conservative treatment often yields suboptimal outcomes, and surgical intervention is typically required to achieve satisfactory recovery and functional restoration.^[1]^ However, surgical management of proximal humeral fractures poses significant challenges for achieving adequate regional analgesia due to the complex innervation of the shoulder and upper arm. Although general anesthesia can provide sufficient intraoperative analgesia, it is associated with systemic risks such as postoperative cognitive dysfunction and respiratory depression. In recent years, the widespread application of ultrasound-guided regional anesthesia has markedly improved the precision and safety of nerve blocks, particularly in anatomically intricate regions such as the neck and shoulder.^[2]^ Ultrasound-guided interscalene brachial plexus block (ISBPB) has become a standard anesthetic approach for proximal humeral surgeries, effectively targeting the C5 to C7 nerve roots. However, its ability to block the C3 to C4 branches of the cervical plexus remains variable and inconsistent across individuals.^[3]^ Studies have reported that approximately 30% of patients experience intraoperative pain in the supraclavicular region despite undergoing ISBPB alone, which is primarily attributed to incomplete coverage of the sensory territory innervated by the superficial cervical plexus.^[4]^ Superficial cervical plexus block (SCPB), targeting the superficial branches of the C2 to C4 nerves, has demonstrated effective analgesia in the anterolateral neck and supraclavicular regions, and is widely used in thyroidectomy and clavicle fracture fixation surgeries.^[5]^ The combined use of ultrasound-guided ISBPB and SCPB can provide satisfactory anesthetic coverage for proximal humerus fracture surgeries. However, this technique may still be associated with complications such as phrenic nerve palsy, Horner syndrome, and nerve injury, which may adversely affect postoperative recovery.^[6]^ Reducing the incidence of such nerve-related complications has become a focal point of interest in clinical anesthesiology practice.

To address the aforementioned challenges, we explored a modified approach combining ultrasound-guided ISBPB with a refined SCPB for anesthesia in proximal humeral fracture surgery. The innovation of this technique lies in the application of hydrodissection along the C5 nerve root, which facilitates the dispersion of local anesthetic within the interscalene space. This method aims to maximize the diffusion of the anesthetic agent while minimizing mechanical trauma to the brachial plexus, thereby enhancing block efficacy and reducing the risk of nerve injury. A safe and effective nerve block technique for proximal humeral fractures can significantly decrease the risk of postoperative pulmonary complications and nerve injury.^[7]^ Moreover, it encourages early participation in rehabilitation, improves overall prognosis and recovery rates, and plays a vital role in the implementation of enhanced recovery after surgery (ERAS) protocols.^[8]^ This study aims to evaluate the clinical value of this novel ultrasound-guided ISBPB combined with a modified SCPB, in comparison with the conventional ISBPB and SCPB technique, in patients undergoing surgery for proximal humeral fractures. Through a prospective randomized controlled trial, we intend to assess the anesthetic efficacy and safety of these 2 approaches. The findings may contribute to optimizing anesthesia strategies for proximal humeral surgeries and provide a new technical pathway for precision regional anesthesia in complex upper limb trauma.

## 2. Materials and methods

### 2.1. Inclusion and exclusion criteria

Inclusion criteria were as follows: patients diagnosed with unilateral proximal humeral fracture based on radiographic imaging, classified as AO/OTA type 11-A, 11-B, or 11-C, and meeting surgical indications; body mass index (BMI) between 18.5 and 29.9 kg/m^2^; American Society of Anesthesiologists (ASA) physical status classification I to III; full understanding of the anesthetic and analgesic procedures, potential complications, and precautions, with signed informed consent for anesthesia and agreement to postoperative follow-up. Exclusion criteria included: Mini-Mental State Examination (MMSE) score < 24; local infection or anatomical abnormality at the puncture site; coagulation disorders; concomitant severe cardiopulmonary disease.

### 2.2. General information

This study was approved by the Institutional Ethics Committee of The Sixth Affiliated Hospital of Xinjiang Medical University (Approved number: LFYLLSC20250425-013), and informed consent was obtained from all patients or their legal representatives. A prospective, randomized, double-blind, controlled trial design was employed. Sample size was calculated using G*Power 3.1 software (Department of Psychology, University of Dusseldorf, Dusseldorf, Germany) (α = 0.05, β = 0.2, effect size *d* = 0.8), resulting in a required total of 60 participants. From January to October 2024, 60 patients undergoing unilateral proximal humeral fracture surgery at our institution who met the inclusion criteria were enrolled and randomly assigned into 2 groups. In the modified group (n = 30), patients received ultrasound-guided ISBPB combined with a modified SCPB. In the control group (n = 30), patients received conventional ISBPB combined with standard SCPB. Both the anesthesiologist performing the block and the investigator collecting outcome data were blinded to group allocation; only the statistician conducting data analysis was aware of the group assignments.

### 2.3. Surgical methods

Preoperative preparation: in accordance with the ERAS protocol, patients fasted for 6 hours and abstained from fluids for 2 hours preoperatively. Upon entering the operating room, standard monitoring was initiated, including electrocardiography, continuous pulse oximetry, and noninvasive blood pressure monitoring.

**Figure 1.**
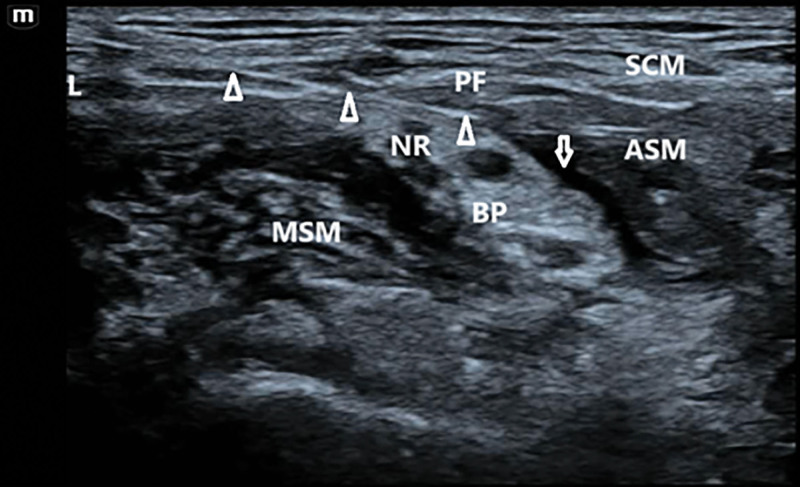
Medial spread of local anesthetic within the interscalene groove. The white triangle indicates the needle tip, while the white arrow shows the direction of local anesthetic spread. ASM = anterior scalene muscle, BP = brachial plexus, L = lateral, MSM = middle scalene muscle, NR = C5 nerve root, PF = prevertebral fascia, SCM = sternocleidomastoid muscle.

**Figure 2.**
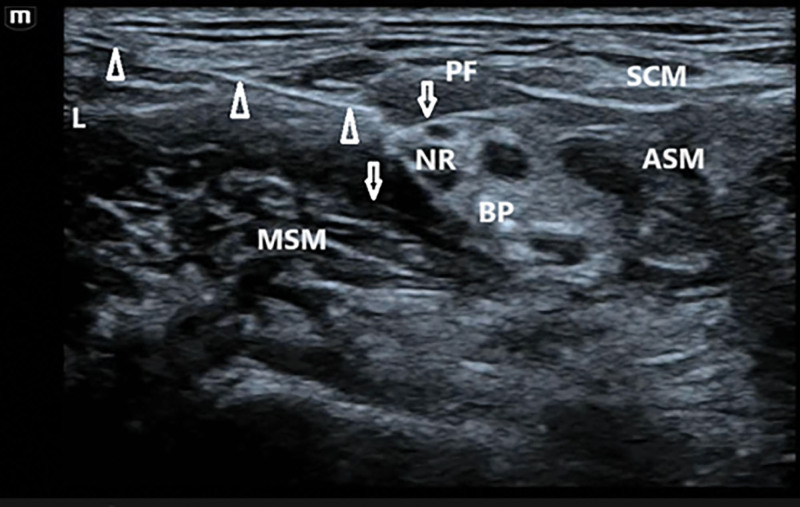
Local anesthetic spread over the C5 nerve root and lateral aspect of the interscalene groove. The white triangle indicates the needle tip, and the white arrow demonstrates the spread of local anesthetic. ASM = anterior scalene muscle, BP = brachial plexus, L = lateral, MSM = middle scalene muscle, NR = C5 nerve root, PF = prevertebral fascia, SCM = sternocleidomastoid muscle.

**Figure 3.**
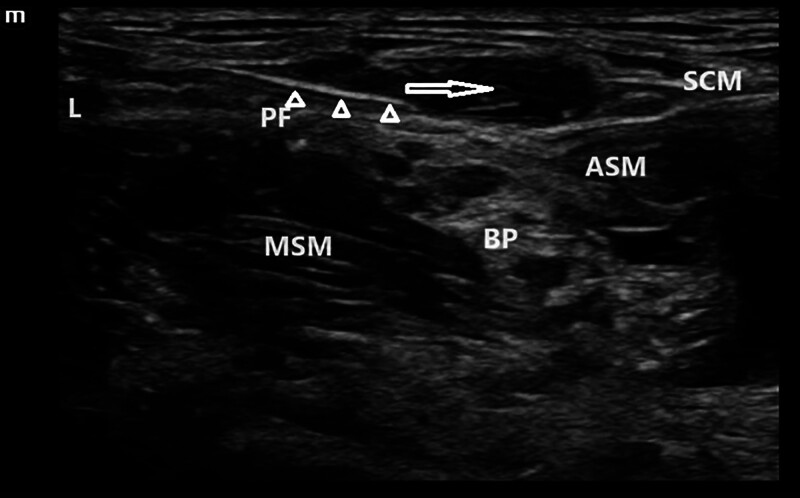
Superficial spread of local anesthetic along the prevertebral fascia. The white triangle indicates the needle tip, and the white arrow shows the trajectory of local anesthetic spread. ASM = anterior scalene muscle, BP = brachial plexus, L = lateral, MSM = middle scalene muscle, NR = C5 nerve root, PF = prevertebral fascia, SCM = sternocleidomastoid muscle.

Modified group – ultrasound-guided ISBPB combined with modified SCPB: the procedure was performed as follows: the patient was positioned supine with the head turned toward the contralateral (nonoperative) side to fully expose the puncture site in the neck. A pre-scan was conducted using ultrasound to identify the anatomical structures of the interscalene brachial plexus and the superficial cervical plexus. Scanning was performed from the infraclavicular region upwards to locate the junction of the prevertebral fascia and the C5 nerve root, identified by the characteristic “fish-mouth sign” on ultrasound^[9]^; after standard aseptic preparation and sterile draping, and with the ultrasound probe covered in a sterile sleeve, an in-plane needle insertion technique was employed. The needle was advanced from lateral to medial, closely along the fascial plane between the C5 nerve root and the prevertebral fascia. A total of 10 mL of 0.4% ropivacaine was injected at the medial side of the C5 root (Fig. 1); the needle was then withdrawn slightly and redirected to the lateral aspect of the C5 root, where an additional 10 mL of 0.4% ropivacaine was administered (Fig. 2); adequate diffusion was confirmed when the local anesthetic circumferentially enveloped the nerve root, with ultrasound evidence of symmetric spread along the interscalene space (diffusion area ≥ 85%), indicating a complete block; finally, the needle was withdrawn to the subcutaneous plane and redirected along the prevertebral fascial plane. An additional 10 mL of 0.4% ropivacaine was injected. The needle trajectory was generally maintained within the level of the anterior scalene muscle. Satisfactory spread of the anesthetic in this plane indicated a successful and complete block (Fig. 3).

Control group – ultrasound-guided conventional ISBPB combined with SCPB: in the control group, the interscalene brachial plexus was identified under ultrasound guidance. After standard skin disinfection and sterile draping, an in-plane needle insertion technique was used, proceeding from lateral to medial. The deep target nerves were blocked first, followed by the superficial targets. If sufficient spacing between nerve roots was noted, the needle was passed through the connective tissue between the nerve roots to the contralateral side of the interscalene groove to facilitate drug spread. A total of 20 mL of 0.4% ropivacaine was injected. Adequate block was confirmed by observing the circumferential diffusion of local anesthetic around the nerves within the interscalene space. For the SCPB, the ultrasound probe was positioned along the lateral border of the sternocleidomastoid muscle. The needle was inserted from the posterolateral direction, and upon reaching the deep fascia at the posterior border of the sternocleidomastoid muscle, 10 mL of 0.4% ropivacaine was injected. Sufficient diffusion within the fascial plane confirmed a successful block.

### 2.4. Observation indicators

The following clinical parameters were recorded and compared between the 2 groups: ASA physical status classification, age, sex, BMI, and duration of surgery. Onset time of sensory block and assessment of block quality: anesthetic efficacy was evaluated using the thermal sensation method. Sensory testing was performed at 1-minute intervals over the dermatomes corresponding to the surgical region on the affected limb. The onset time of anesthesia was defined as the time at which the patient reported loss of temperature sensation in the target area. If no sensory block was achieved within 30 minutes, the block was considered a failure, and general anesthesia was administered. The duration of sensory block was defined as the time from completion of the nerve block to the first report of postoperative pain. The block quality was graded as excellent, good, or poor. Excellent: complete block coverage, patient remained pain-free and calm with satisfactory muscle relaxation throughout surgery, no requirement for supplemental analgesics or sedatives. Good: incomplete block, patient showed mild discomfort or facial expression of pain during surgery and the surgical procedure was successfully completed with supplemental medication. The supplemental medication protocol was propofol 2 mg/kg administered as slow intravenous injection to induce loss of consciousness, followed by continuous intravenous infusion at 5 mg/kg/h. Poor: inadequate block, even with supplemental medication, patient could not tolerate the procedure and conversion to general anesthesia was necessary. The general anesthesia protocol was as follows: induction: propofol 2 mg/kg, sufentanil 0.4 µg/kg, and rocuronium 0.6 mg/kg were administered intravenously. After achieving mandibular muscle relaxation, endotracheal intubation was performed. Maintenance: continuous infusion of propofol at 5 mg/kg/h with remifentanil 6 µg/kg/h. Incidence of block-related complications: the occurrence of Horner syndrome, phrenic nerve palsy, and brachial plexus injury was recorded. Horner syndrome was diagnosed by the presence of ipsilateral miosis, ptosis, and facial flushing, indicating sympathetic blockade. Phrenic nerve function was assessed by comparing diaphragmatic excursion before and after the block. Diaphragmatic excursion was evaluated using M-mode ultrasound by measuring the vertical distance of diaphragmatic displacement between end-inspiration and end-expiration. A reduction in diaphragmatic movement ≤ 25% was defined as mild paresis, 25 to 75% as moderate paresis, and ≥ 75% as severe paresis.^[10]^ Nerve injury evaluation: the incidence of nerve-related complications, including sensory deficits, neuropathic pain, and neurogenic edema, was documented. Patient satisfaction within 24 hours postoperatively: satisfaction was assessed based on 2 primary factors: the incidence of pain (defined as VAS score > 3) and the rate of anesthesia-related complications causing patient discomfort.

### 2.5. Statistical analysis

Data were analyzed using SPSS version 19.0 (IBM Corp., Armonk). Categorical variables were compared using the chi-square test, while continuous variables were expressed as mean ± standard deviation (SD) and analyzed using independent samples *t*-tests. A *P*-value < .05 was considered statistically significant.

## 3. Results

### 3.1. Patient baseline characteristics

A total of 60 patients undergoing unilateral proximal humeral fracture surgery were enrolled and randomly assigned to either the modified group (n = 30; 18 males, 12 females) or the control group (n = 30; 19 males, 11 females). The mean age in the modified group was 40.2 years, with a mean BMI of 26.1 kg/m^2^; ASA physical status classification ranged from I to II. In the control group, the mean age was 39.3 years, with a mean BMI of 25.3 kg/m^2^, and ASA classification was also I to II. There were no statistically significant differences between the 2 groups with respect to sex distribution, age, BMI, ASA classification, or fracture staging (*P* > .05) (Table 1). These findings indicate that both groups were comparable in terms of baseline demographic and surgical characteristics, thereby supporting the validity of randomization and the reliability of subsequent outcome comparisons.

**Table 1:** Baseline characteristics of patients.

Variable	Modified group	Control group	*t*	*P*
Sex (male/female)	18/12	19/11	0.071	.791
Age (yr)	40.2 ± 11.4	39.3 ± 12.3	0.295	.769
Body mass index (kg/m^2^)	26.1 ± 7.2	25.3 ± 7.4	0.892	.376
ASA classification (I/II)	17/13	16/14	0.067	.795

### 3.2. Comparison of anesthetic block quality and duration of sensory block between the 2 groups

To further evaluate the impact of the 2 anesthetic techniques on block quality and duration of sensory block, additional analyses were performed on operative time, sensory block quality, and duration of anesthesia. The mean operative time and duration of sensory block in the modified group were 93 minutes and 6.2 hours, respectively, with most patients demonstrating an excellent sensory block. In the control group, the mean operative time and sensory block duration were 94 minutes and 6.3 hours, respectively, with similarly favorable block quality. There were no statistically significant differences between the modified and control groups in terms of operative time, sensory block quality, or duration of anesthesia (*P* > .05) (Table 2).

**Table 2:** Comparison of anesthetic block quality and duration of sensory block.

Variable	Modified group	Control group	*t*	*P*
Duration of surgery (min)	93 ± 8.9	94 ± 8.6	0.430	.669
Block quality	30/0	30/0	–	–
Duration of sensory block	6.2 ± 1.9	6.3 ± 1.2	0.369	.714

#### 3.2.1. Comparison of onset time of anesthesia and incidence of Horner syndrome between the 2 groups

The mean onset time of anesthesia in the modified group was 9.1 ± 0.2 minutes, which was significantly longer than that of the control group (3.4 ± 0.1 minutes). This prolongation may be attributed to the increased precision of the modified block technique and the specific diffusion characteristics of the local anesthetic. In terms of complications, the incidence of Horner syndrome was significantly lower in the modified group (33%) compared to the control group (66%) (*P* < .05) (Table 3). These findings suggest a notable advantage of the modified technique in reducing the occurrence of Horner syndrome, potentially related to differences in the diffusion pattern of the local anesthetic and the targeted anatomical plane of the block.

**Table 3:** Comparison of onset time of anesthesia and incidence of Horner syndrome.

Variable	Modified group	Control group	*t*	*P*
Onset time of anesthesia	9.1 ± 0.2	3.4 ± 0.1	53.434	.001
Incidence of Horner syndrome (n, %)	10, 33%	20, 66%	6.667	.010

### 3.3. Comparison of diaphragmatic paresis severity at 30 minutes postoperatively between the 2 groups

At 30 minutes postoperatively, 10 patients (33%) in the modified group exhibited mild diaphragmatic paresis, and 6 patients (20%) experienced moderate paresis. Notably, no cases of severe paresis were observed in this group. In contrast, in the control group, 15 patients (50%) developed mild paresis, 11 patients (37%) had moderate paresis, and 4 patients (13%) exhibited severe diaphragmatic paresis. The overall incidence and severity of diaphragmatic paresis were significantly lower in the modified group compared to the control group (*P* < .05) (Table 4). Importantly, the modified technique completely avoided cases of severe paresis. These findings suggest a clear advantage of the modified block approach in reducing the risk of postoperative diaphragmatic paralysis.

**Table 4:** Comparison of diaphragmatic paresis severity at 30 min postoperatively.

Severity of diaphragmatic paresis	Modified group	Control group	*t*	*P*
Mild paresis (n, %)	10, 33%	15, 50%	1.714	.019
Moderate paresis (n, %)	6, 20%	11, 37%	2.052	.015
Severe paresis (n, %)	0, 0%	4, 13%	2.411	.012

### 3.4. Comparison of the incidence of nerve injury within 24 hours postoperatively

At 24 hours postoperatively, the incidence of nerve injury was significantly lower in the modified group compared to the control group (*P* < .05). Notably, sensory disturbances were effectively avoided in the modified group, whereas 3 patients (10%) in the control group reported sensory deficits. This reduction may be attributed to the improved precision of the modified block technique and its enhanced protective effect on neural structures (Table 5).

**Table 5:** Comparison of nerve injury incidence within 24 h postoperatively.

Variable	Modified group	Control group	*t*	*P*
Sensory disturbance (n, %)	0, 0%	3, 10%	1.404	.024
Neuropathic pain (n, %)	0, 0%	0, 0%	–	–
Neurogenic edema (n, %)	0, 0%	0, 0%	–	–

### 3.5. Comparison of patient satisfaction within 24 hours postoperatively

Patient satisfaction at 24 hours postoperatively was significantly higher in the modified group than in the control group (*P* < .05). The satisfaction rate in the modified group was 97%, compared to 87% in the control group. This improvement is likely due to a lower incidence of complications and better pain control. Although both groups had 1 patient (3.3%) who reported experiencing postoperative pain (defined as VAS score > 3), the overall satisfaction was higher in the modified group due to the reduced rate of block-related complications. In the modified group, no patients reported dissatisfaction due to complications, while in the control group, 3 patients (10%) reported that complications negatively affected their postoperative experience (Table 6). These findings further highlight the advantages of the modified technique in enhancing patient satisfaction.

**Table 6:** Comparison of patient satisfaction and postoperative discomfort within 24 h.

Variable	Modified group	Control group	*t*	*P*
Pain experience (n, %)	1, 3.3%	1, 3.3%	0.000	1.000
Complication-related discomfort (n, %)	0, 0%	3, 10%	1.404	.024
Patient satisfaction (n, %)	97%	87%	0.873	.035

## 4. Discussion

The anesthetic efficacy in proximal humeral fracture surgery plays a critical role in ensuring the smooth conduct of the procedure and facilitating optimal postoperative recovery. Therefore, identifying a safe and effective anesthesia technique is essential.^[11]^ This study aimed to evaluate the efficacy and safety of ultrasound-guided ISBPB combined with a modified SCPB in patients undergoing surgery for proximal humeral fractures. The results demonstrated that, compared with the conventional ISBPB combined with standard SCPB, the modified technique provided comparable anesthetic efficacy while significantly reducing the incidence of complications such as phrenic nerve palsy, Horner syndrome, and nerve injury. Additionally, patient satisfaction scores were markedly higher in the modified group. These findings carry important clinical implications and offer new insights into the selection of regional anesthesia techniques for proximal humeral fracture repair.

Historically, single-shot ISBPB alone has been used for proximal humeral fracture surgery. However, its analgesic effect is often inadequate, necessitating the use of supplemental analgesics and sedatives. One of the main reasons for incomplete anesthesia is that the surgical incision commonly involves the supraspinatus and infraspinatus muscles around the acromion – regions innervated by the supraclavicular nerves. These nerves originate from the cervical plexus at the root of the neck, traverse the acromial end of the scapula, and enter the joint capsule to innervate the supraspinatus and infraspinatus muscles. Therefore, SCPB is essential to achieve adequate anesthesia coverage of the supraclavicular nerve territory. A combined approach using interscalene and SCPB is thus necessary to ensure complete anesthetic coverage in proximal humeral fracture surgery. In the present study, both the modified and control groups achieved satisfactory anesthetic block quality, with no statistically significant difference between them. However, the onset time of anesthesia in the modified group was significantly longer than that in the control group. This delay may be attributed to the modified technique’s reliance on gradual diffusion of local anesthetic through the interscalene space into the neural sheath, leading to a slower onset.

A significant advantage observed in the modified group was the reduced incidence of nerve injury. The modified technique involves targeted puncture at the distal, superior, and proximal aspects of the C5 nerve root, combined with hydrodissection to facilitate controlled spread of local anesthetic along the interscalene groove. This approach contributes to the lower incidence of nerve injury observed, suggesting a reduced risk of direct needle trauma to neural structures through precise needle positioning, which consequently promotes better postoperative functional recovery. Anatomically, the phrenic nerve originates from the anterior rami of the C3 to C5 spinal nerves. It descends medially along the anterior surface of the anterior scalene muscle, passing deep to the prevertebral fascia, and courses between the subclavian artery and vein before entering the thoracic cavity. Since inadvertent phrenic nerve blockade may lead to diaphragmatic paresis and respiratory compromise,^[12,13]^ potentially increasing the risk of postoperative pulmonary complications, it is essential to minimize this risk whenever possible. One of the key findings of this study is that the modified block technique significantly reduced the incidence of diaphragmatic paresis, especially severe paresis. This may be explained by the distinct diffusion patterns of local anesthetic in the modified approach. In the interscalene groove, local anesthetic in the modified group spreads caudally within the fascial compartment without penetrating deep into the anterior scalene muscle. Additionally, in the modified SCPB, the local anesthetic spreads within the superficial layer of the deep cervical fascia and generally does not reach the deep plane of the anterior scalene muscle. As a result, the risk of phrenic nerve blockade is greatly reduced, contributing to preserved respiratory function and a lower incidence of pulmonary complications.^[14,15]^

Moreover, the modified technique also significantly reduced the incidence of Horner syndrome. Horner syndrome results from blockade of the cervical sympathetic chain,^[16]^ which lies anterolateral to the cervical vertebrae, running longitudinally along the superficial surface of the longus capitis and longus colli muscles, deep to the prevertebral fascia, posterior to the carotid sheath, and medial to the vagus nerve. Blockade of this structure may manifest as ipsilateral ptosis, miosis, conjunctival injection, blurred vision, and facial flushing. Although typically self-limiting with the metabolism of local anesthetic,^[6]^ the syndrome can cause patient distress and facial discomfort, and is therefore best avoided. The reduced incidence of Horner syndrome in the modified group may be attributed to the altered diffusion pattern of the anesthetic. In the modified interscalene block, the local anesthetic spreads inferiorly along the interscalene groove, rather than medially toward the anterior scalene muscle, thereby reducing the likelihood of sympathetic chain involvement. Furthermore, in the modified SCPB, the spread of local anesthetic remains within the superficial layer of the deep cervical fascia, without reaching the level of the anterior scalene muscle, further decreasing the chance of unintentional sympathetic blockade.

In surgeries for proximal humeral fractures, both the modified and control groups provided effective intraoperative anesthesia that allowed for smooth completion of the procedures. While the overall block efficacy was comparable between the 2 groups, the modified group demonstrated significantly lower incidences of diaphragmatic paresis, Horner syndrome, and nerve injury, along with higher patient satisfaction – factors that contribute positively to postoperative recovery. The key innovation of this study lies in the use of hydrodissection around the C5 nerve root, which facilitates optimal diffusion of local anesthetic within the interscalene groove. This technique helps prevent mechanical injury to the brachial plexus while achieving satisfactory block efficacy. Furthermore, in the modified SCPB, the needle is introduced into the deep cervical fascia above the middle scalene muscle. Given that the supraclavicular nerves typically traverse the anterosuperior aspect of the middle scalene muscle, this approach allows for more effective blockade of the supraclavicular nerve, thereby enhancing anesthetic coverage. Anatomically, the phrenic nerve descends medially from the superolateral border of the anterior scalene muscle, while the cervical sympathetic chain runs longitudinally along the superficial surface of the longus capitis and longus colli muscles, deep to the prevertebral fascia. In the modified technique, ultrasound confirmed that the spread of local anesthetic did not reach the superficial plane of the anterior scalene muscle, thereby minimizing the risk of phrenic nerve paralysis and Horner syndrome. Although the onset time of anesthesia was slightly prolonged in the modified group, there was no significant difference in overall block efficacy between the groups, indicating that the modified technique improves safety without compromising anesthetic performance.

This study has several limitations. First, the sample size was relatively small, which may limit the generalizability and statistical power of the findings. Second, only 0.4% ropivacaine was used in this study; other concentrations and types of local anesthetics were not evaluated, nor were larger population subsets analyzed. Additionally, the study did not include long-term follow-up to assess outcomes such as chronic pain, functional recovery, or long-term safety of the anesthetic techniques.

In conclusion, the use of ultrasound-guided ISBPB combined with a modified SCPB provides effective anesthesia for proximal humeral fracture surgery while significantly reducing the incidence of phrenic nerve palsy, Horner syndrome, and nerve injury. It also enhances patient satisfaction. Given these benefits, this modified technique holds promise for broader clinical application and offers a safer and more comfortable anesthetic option for patients undergoing proximal humeral fracture repair.

## Author contributions

**Conceptualization**: Gang Liu.

**Data curation**: Gang Liu.

**Formal analysis**: Xiaoxuan Du.

**Funding acquisition**: Feng Song.

**Investigation**: Lei Gao.

**Methodology**: Xiaoxuan Du.

**Project administration**: Feng Song.

**Resources**: Lei Gao.

**Software**: Xiaoxuan Du.

**Supervision**: Lei Gao, Feng Song.

**Validation**: Wei Wang.

**Visualization**: Wei Wang.

**Writing – original draft**: Gang Liu.

**Writing – review & editing**: Wei Wang, Feng Song.
